# Sterically Selective [3 + 3] Cycloaromatization in the On-Surface Synthesis of Nanographenes

**DOI:** 10.1021/acsnanoscienceau.3c00062

**Published:** 2023-12-22

**Authors:** Amogh Kinikar, Xiao-Ye Wang, Marco Di Giovannantonio, José I. Urgel, Pengcai Liu, Kristjan Eimre, Carlo A. Pignedoli, Samuel Stolz, Max Bommert, Shantanu Mishra, Qiang Sun, Roland Widmer, Zijie Qiu, Akimitsu Narita, Klaus Müllen, Pascal Ruffieux, Roman Fasel

**Affiliations:** 1 Empa, Swiss Federal Laboratories for Materials Science and Technology, 8600 Dübendorf, Switzerland; 2 Max Planck Institute for Polymer Research, 55128 Mainz, Germany; 3 State Key Laboratory of Elemento-Organic Chemistry, College of Chemistry, Nankai University, Tianjin 300071, China; 4 Department of Chemistry, Johannes Gutenberg-Universität Mainz, 55128 Mainz, Germany; 5 Department of Chemistry, Biochemistry and Pharmaceutical Sciences, University of Bern, 3012 Bern, Switzerland; 6 Institute of Condensed Matter Physics, Station 3, EPFL, 1015 Lausanne, Switzerland

**Keywords:** on-surface synthesis, cycloaromatization, STM, nc-AFM, selectivity, steric protection

## Abstract

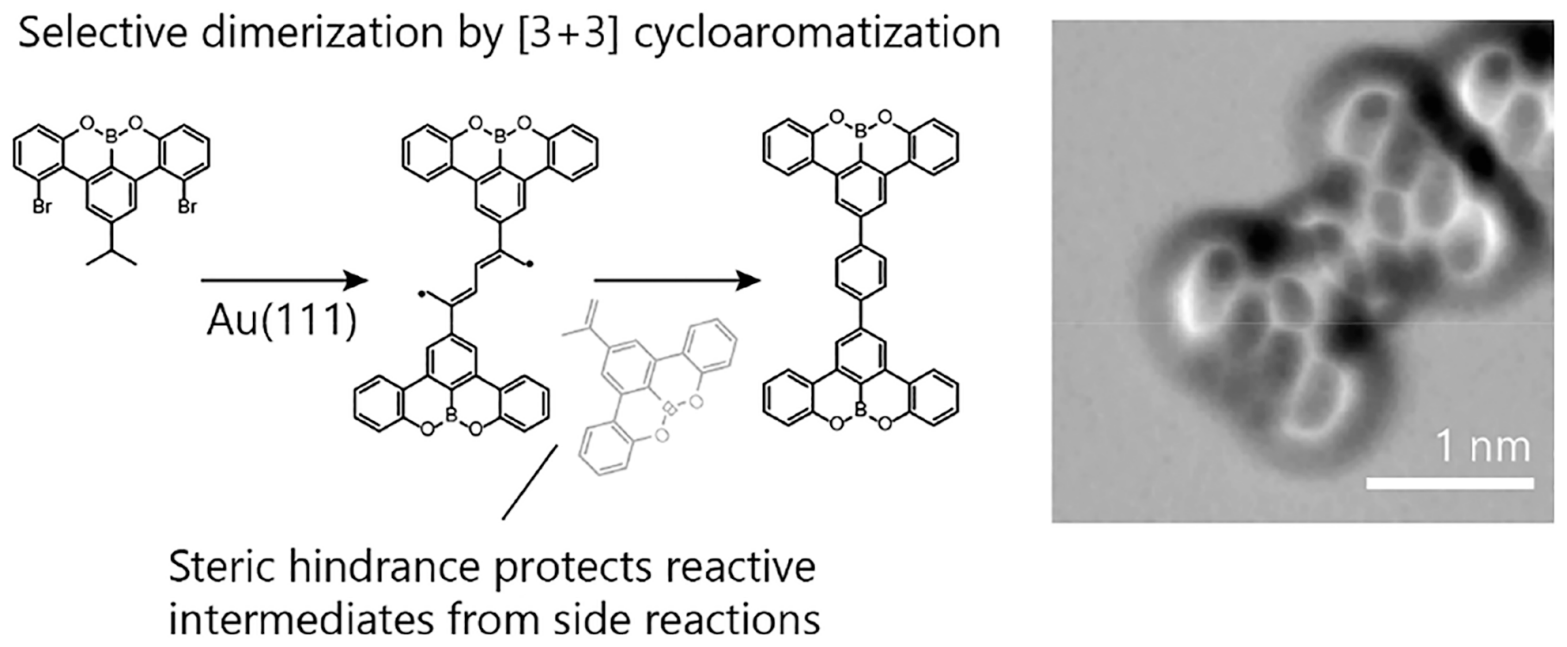

Surface-catalyzed reactions have been used to synthesize carbon nanomaterials with atomically predefined structures. The recent discovery of a gold surface-catalyzed [3 + 3] cycloaromatization of isopropyl substituted arenes has enabled the on-surface synthesis of arylene-phenylene copolymers, where the surface activates the isopropyl substituents to form phenylene rings by intermolecular coupling. However, the resulting polymers suffered from undesired cross-linking when more than two molecules reacted at a single site. Here we show that such cross-links can be prevented through steric protection by attaching the isopropyl groups to larger arene cores. Upon thermal activation of isopropyl-substituted 8,9-dioxa-8a-borabenzo[*fg*]tetracene on Au(111), cycloaromatization is observed to occur exclusively between the two molecules. The cycloaromatization intermediate formed by the covalent linking of two molecules is prevented from reacting with further molecules by the wide benzotetracene core, resulting in highly selective one-to-one coupling. Our findings extend the versatility of the [3 + 3] cycloaromatization of isopropyl substituents and point toward steric protection as a powerful concept for suppressing competing reaction pathways in on-surface synthesis.

## Introduction

When embarking on the synthesis of novel materials, it is imperative to consider the intricacies of the synthetic protocol and, notably, the selectivity of all of the involved reaction steps. Beyond merely impacting reaction yields, suboptimal selectivity can give rise to defects within the targeted material that potentially induce unintended properties. In the context of atomically precise carbon nanomaterials, the preferred approach often revolves around what is nowadays called “on-surface synthesis”.^1,2^ This methodology involves the execution of discerning surface-catalyzed reactions on meticulously designed molecular precursors under ultrahigh vacuum conditions. The fundamental tenet behind achieving selectivity in on-surface synthesis hinges on either engendering a specific class of radicals that homocouple (in the case of intermolecular reactions) or harnessing the spatial proximity of reactive moieties (for intramolecular reactions).^3^ Notably, our recent research has unveiled the capacity of gold surfaces in catalyzing the cycloaromatization of isopropyl substituents on aryl cores, resulting in a [3 + 3] cyclization wherein two isopropyl substituents merge to form a phenyelene ring.^4^

**Figure 1:**
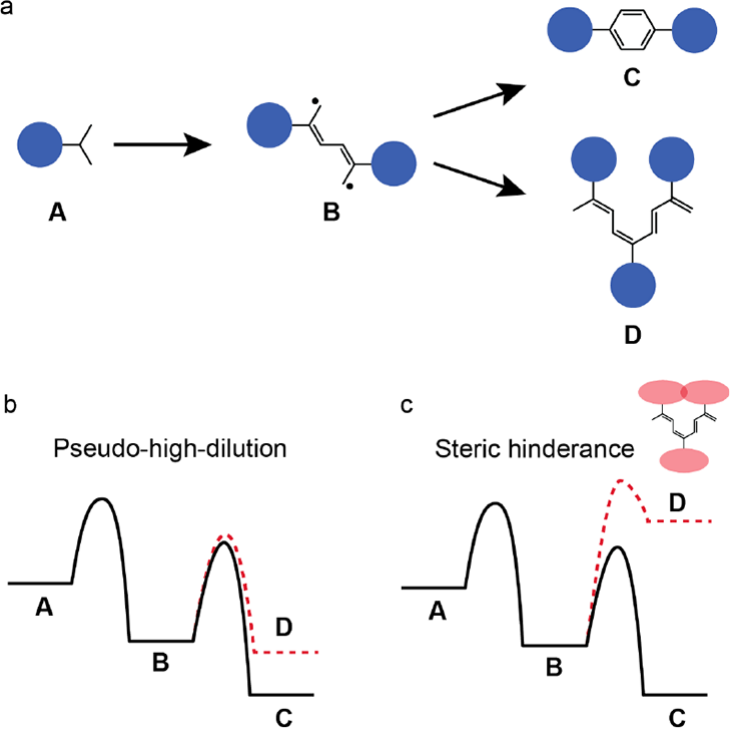
(a) Chemical sketch showing the [3 + 3] cycloaromatization of isopropyl substituents in **A** to yield **C**, via the highly reactive intermediate **B**. The radical sites in **B** can be attacked by a third molecule leading to a side reaction yielding **D**. (b) The selectivity of the reaction toward target **C** can be enhanced under pseudohigh-dilution conditions, i.e. by depositing a low flux of the molecule on the hot substrate. (c) Installing isopropyl groups on wider aryl cores sterically prohibits the synthesis of **D**, thereby increasing the selectivity of the reaction toward the target **C**.

Nevertheless, our investigations also unveiled the occurrence of side-reactions resulting from three or more isopropyl substituents reacting at a given site, thereby diminishing both the selectivity and the overall reaction yield. These undesirable side reactions stem from the pronounced reactivity of an intermediate in the reaction pathway (labeled as **B** in Figure 1a) being susceptible to attack by a third molecular species. Given that the formation of a C–C bond is an irreversible step under the current reaction conditions, a straightforward strategy to mitigate these side reactions involves preventing the intrusion of the third molecule into the reaction intermediates. Previously, we demonstrated that one viable approach to achieve this goal is the employment of pseudohigh-dilution conditions.^5^ This entails the deposition of molecular precursors onto the gold surface at an exceedingly low flux while maintaining temperatures higher than those necessary to activate the reaction (Figure 1b). Here selectivity over the reaction products is achieved by significantly enhancing the likelihood of the reactive intermediate undergoing the desired cyclization reaction, far surpassing the probability of a third molecule impinging upon the intermediate due to the low precursor flux.

In this study, we showcase that similar control over the reaction products can be achieved through judicious introduction of steric hindrance (Figure 1c). By strategically installing isopropyl substituents onto wider aryl cores, the reaction is sterically confined to involve only two molecules. To elucidate this phenomenon, we provide comprehensive characterizations of the reaction products using scanning tunneling microscopy (STM) and non-contact atomic force microscopy (nc-AFM), complemented by density functional theory (DFT) simulations, thereby unraveling the underlying factors contributing to the remarkable one-to-one coupling selectivity. Furthermore, our investigation is enriched by temperature-programmed X-ray photoelectron spectroscopy (TP-XPS) measurements, which furnish additional insights into the intricate dynamics of the cycloaromatization process.

## Results and Discussion

### Selective Dimerization by [3 + 3] Cycloaromatization

**Scheme 1:**
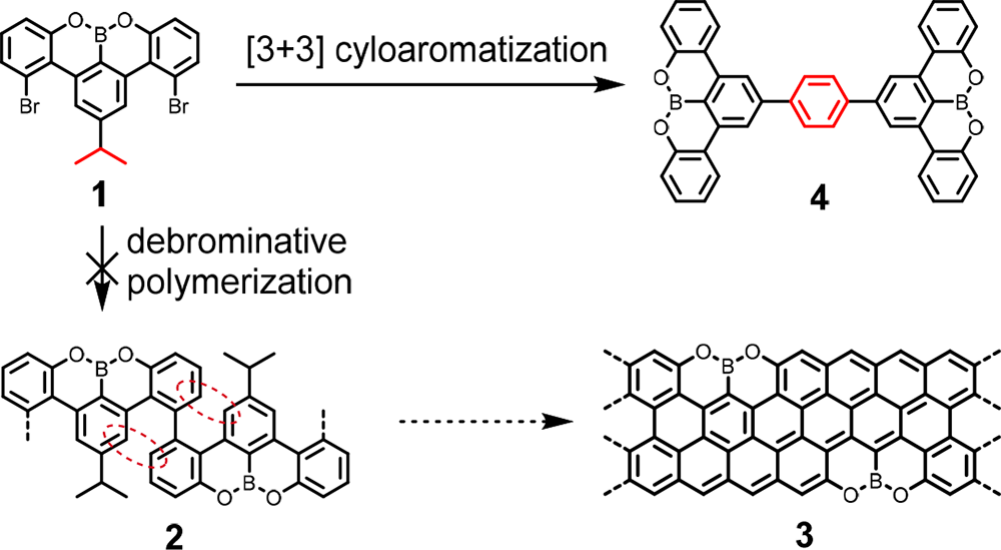
Expected and Observed On-Surface Reaction of 1 on Au(111)

The molecule 4,13-dibromo-2-isopropyl-8,9-dioxa-8*a*-borabenzo[fg]tetracene (**1**) (Scheme 1) was synthesized as a potential precursor for the synthesis of the OBO-ZGNR **3** by cyclodehydrogenation of the polymer resulting from debrominative homocoupling, **2**. However, **2**, could not be achieved because the access to the radical sites generated by debromination is sterically hindered (red dashed ellipses, Scheme 1). As we will show in the subsequent discussion, **1** instead undergoes highly selective dimerization via [3 + 3] cycloaromatization toward **4** (Scheme 1).

**Figure 2:**
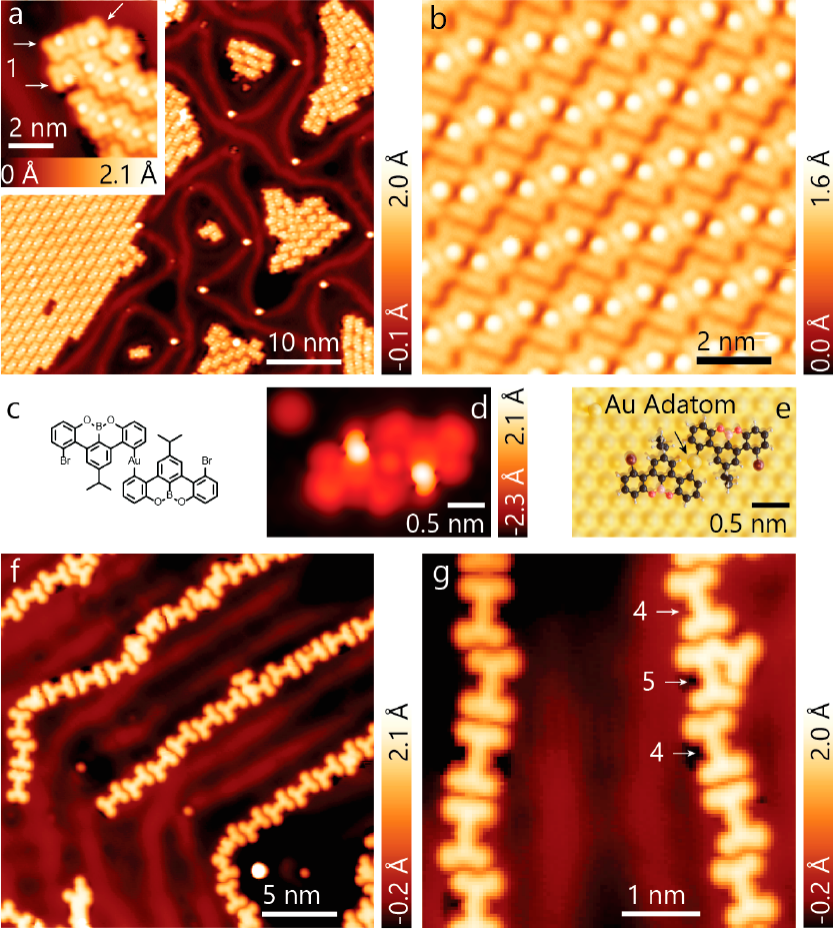
Achieving high selectivity of [3 + 3] cycloaromatization. (a) STM images acquired on the Au(111) surface after deposition of **1** and subsequent annealing to 160 °C show the formation of large self-assembled islands. (Tunneling parameters: *V* = 0.3 V, *I* = 10 pA.) Inset: A few still fully brominated molecules of **1** (indicated by the arrows) are observed in the smaller islands (*V* = −0.5 V and *I* = 10 pA). (b) STM image acquired inside one of the self-assembled islands, revealing that these are composed of dimers (*V* = −0.5 V, *I* = 10 pA). (c) The dimers consist of two singly debrominated molecules of **1** bound through a Au adatom. (d,e) Simulated STM image (d, at *V* = −0.52 V) of the DFT-optimized geometry of the proposed dimer structure shown in (e). (f) Annealing the surface to 200 °C induces the [3 + 3] cycloaromatization yielding **4** (V = −0.1 V, I = 100 pA). (g) Among the minor byproducts, the molecule labeled **5** is most often observed (*V* = −0.1 V, *I* = 100 pA).

Precursor **1** was synthesized in four steps from commercially available 4-isopropylaniline (for details, see the Supporting Information). **1** was deposited onto a clean Au(111) single-crystal surface held at room temperature under ultrahigh vacuum conditions. The subsequent sample annealing at 160 °C triggered homolytic cleavage of the C–Br bond. TP-XPS maps (shown in Figure 4 and discussed below) reveal an almost complete debromination of **1** at 160 °C, which however did not afford the initially targeted structure **2** due to the steric repulsion between the aromatic hydrogen atoms of the molecular cores (red areas in Scheme 1).^6,7^ Instead, STM images of the surface obtained upon cooling to 5 K show large islands consisting of dimers (Figure 2a,b).

Radical sites, being highly reactive, can undergo a variety of reactions, such as metalation with Au adatoms, bromination with chemisorbed Br atoms, or passivation with H atoms. Previous work has shown a temperature-dependent equilibrium on Au(111) between debrominated products and brominated precursors.^8^ Presently, heating the surface to 160 °C did not lead to the complete desorption of Br atoms chemisorbed on the Au surface, as evidenced by the distorted herringbone reconstruction (Figure 2a). We therefore speculate that, after the annealing step, cooling of the sample for imaging caused partial rebromination from the available bromine atoms that are chemisorbed on the gold surface (see Figure 4f). We propose that the dimers result from a competition between the two reversible reactions that are possible on the surface: formation of a C–Br bond or formation of a C–Au bond. The image shows that within a dimer, two precursor molecules are bound to one atomic species on the surface. This clearly cannot be a Br as it is monovalent and therefore, it must be an Au adatom. Second, we do not see the formation of organo-metallic chains, but simply the formation of dimers. Thus, the second radical site on the precursor molecule must be passivated with a monovalent species, i.e., by Br leading to the proposed structure shown in Figure 2c. Simulated STM images of the proposed organometallic dimers, optimized using DFT, agree well with the experimental findings (Figure 2c–e).

A striking change occurs upon further annealing the surface to 200 °C (Figure 2f). The self-assembled organometallic dimers have given way to self-assembled chains of molecules of **4** with a distinctive ‘dog-bone’ shape (Figure 2f). As we will show below, these dog-bone-shaped molecules are covalently linked dimers that have undergone the intermolecular [3 + 3] cycloaromatization of their isopropyl substituents. The molecules are organized into chains as a results of the hydrogen bonding between the OBO-substitutions in the benzotetracene core. We find that 77 ± 6% of the precursor units have been consumed in the formation of **4** (see Supporting Information S1). This shows that highly selective one-to-one coupling using the [3 + 3] cycloaromatization reaction has been achieved on the Au(111) surface.

### Chemical Structure and Formation Mechanism of **4**

**Figure 3:**
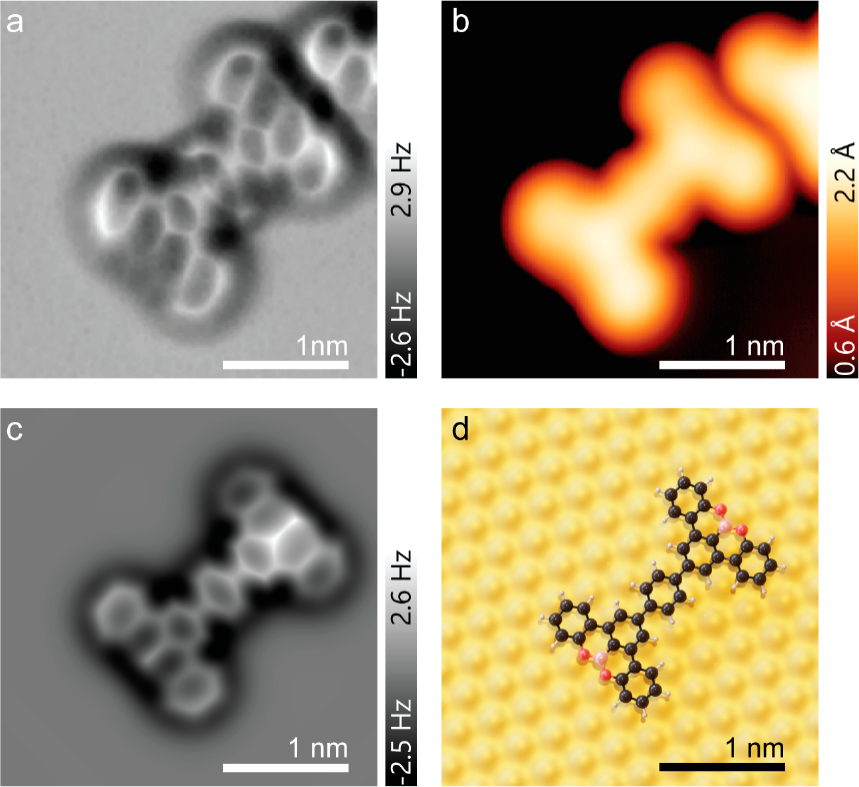
Chemical structure of 4 determined by nc-AFM. (a) Bond-resolved nc-AFM image acquired over a molecule of **4** resolves the two aryl subunits and the phenylene ring coupling them (feedback switched off over the Au surface at −5 mV, 150 pA; the tip was lifted up by Δ*z* = 235 pm, and the frequency shift was measured in constant-height mode). (b) STM image of the same molecule (*V* = −0.1 V, *I* = 150 pA). (c) Simulated nc-AFM image, using the probe-particle model, of **4** on an Au(111) slab with DFT-optimized geometry, is in good qualitative agreement with the experimental results. (d) Top view of the optimized geometry of **4** on Au(111).

The nc-AFM in Figure 3a confirms the chemical structure of **4**, clearly resolving its two benzotetracene cores and the phenyelene linker. Although the latter appears wider, it is in agreement with previous reports on molecules containing similar *para* phenyelene linkers,^9−11^ with the apparent widening attributed to bending of the CO molecule on the nc-AFM tip^12^ (see Supporting Information S2). DFT geometry optimization determined the minimum-energy adsorption conformation of **4** on the Au(111) surface (Figure 3d). Simulated nc-AFM images^13,14^ of this structure (Figure 3c) match the experimental image. The DFT simulation can also explain the difference in contrast seen on the two zigzag edges of the molecule. When the oxygen atoms are adsorbed over Au atoms they appear darker; when they are adsorbed over a bridge site they appear brighter. Evidently the presence of a Au atom directly underneath the oxygen atoms pulls them closer to the substrate.

**Scheme 2:**
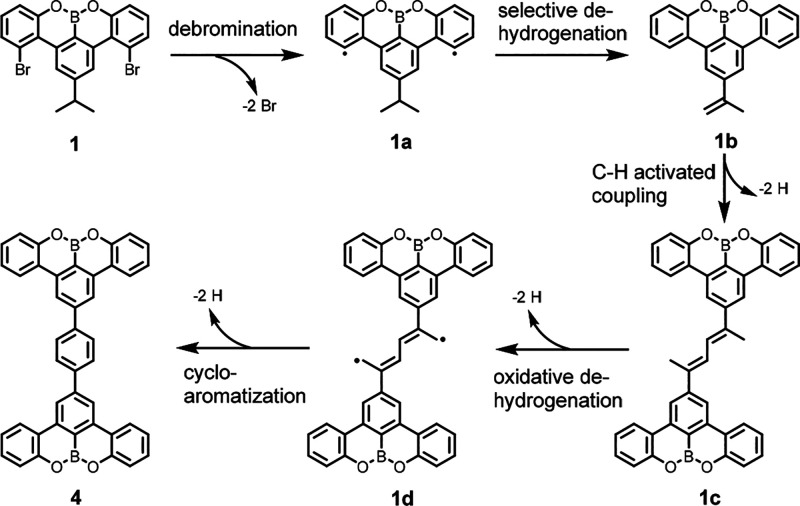
Proposed Reaction Pathway for the On-Surface Synthesis of 4

Based on the previously reported [3 + 3] cycloaromatization mechanism,^4^ we propose a reaction pathway for the formation of **4** (Scheme 2): C–Br bond cleavage occurs below 160 °C on the Au(111) surface, and annealing to 200 °C triggers the cycloaromatization. This process is initiated by the dehydrogenation of the isopropyl-substituent into an isopropenyl-group (**1b**), leading to the formation of **1c** through C–H activated coupling at the CH_2_ site. Further oxidative dehydrogenation forms **1d** which undergoes cycloaromatization to finally afford **4**.

Additionally, ten H atoms are released per molecule of **4** during cycloaromatization, stochastically passivating the four radical sites that were generated by debromination.^15^ Among the few side products, trimeric molecule **5** is frequently observed and contains a preformed cycloaromatization dimer (see Supporting Information S3). In **5**, the site of the cleaved C–Br bond on the cycloaromatization product is covalently coupled to a third molecule at its isopropyl substituent site. This reaction is likely to occur only if the radical site generated by debromination has not been passivated by hydrogen (mechanistic details are shown in Supporting Information S4). The presence of **5** evidences that [3 + 3] cycloaromatization and radical site passivation occur simultaneously. It is the presence of the radical sites generated by debromination that leads to side reactions, limiting the yield of **4** to ∼80%.

### Selective One-to-One Coupling Achieved by Steric Protection

**Scheme 3:**
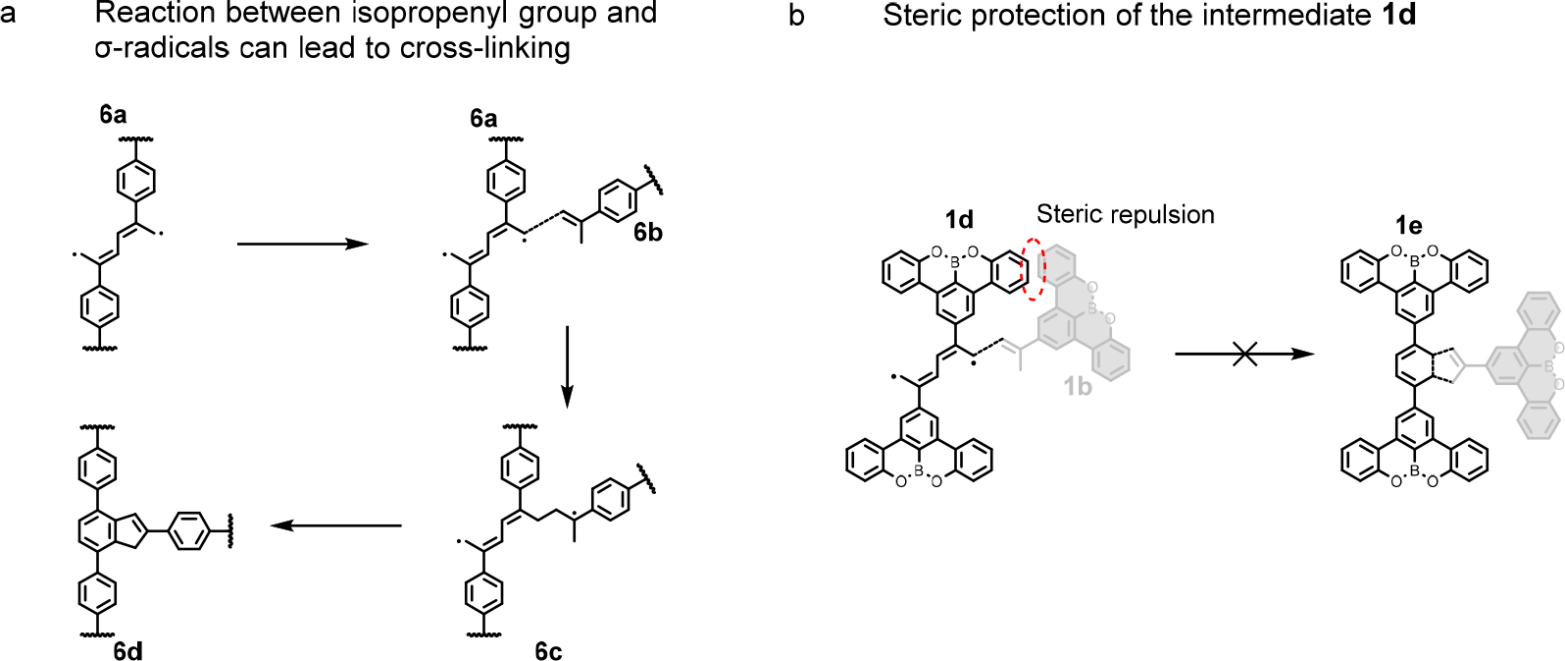
Steric Protection from Cross-Linking (a) Proposed reaction pathway leading to cross-linking. (b) Steric protection afforded by the large OBO-doped benzotetracene core prevents the coupling of three or more molecules.

Reactions involving more than two molecules at the isopropyl site are never seen on the surface; the yield of dimerization arising from the [3 + 3] cycloaromatization is thus 100%. This safeguard is attributed to the steric protection from the large benzotetracene core, which is not present when the isopropyl group is affixed to smaller arenes.^4,16^ Supporting this, the key stages of the cross-linking reaction pathway are outlined in Scheme 3a. **6a**, a cycloaromatization intermediate, is highly reactive. Without steric protection, radicals in **6a** can be attacked by the CH_2_ group of another molecule, **6b**. This leads to three molecules coupling at the isopropyl site, forming cross-linked polymers **6c** and eventually yielding **6d**. In our case (Scheme 3b), the large, planar, OBO-doped benzotetracene core and the 2D confinement onto the surface contribute to shield the radical sites in **1d** against additional coupling reactions. Steric protection^17^ can prevent unwanted cross-linking, extending the utility of surface-catalyzed [3 + 3] cycloaromatization.

### Kinetics of [3 + 3] Cycloaromatization from Temperature-Programmed X-ray Photoelectron Spectroscopy

**Figure 4:**
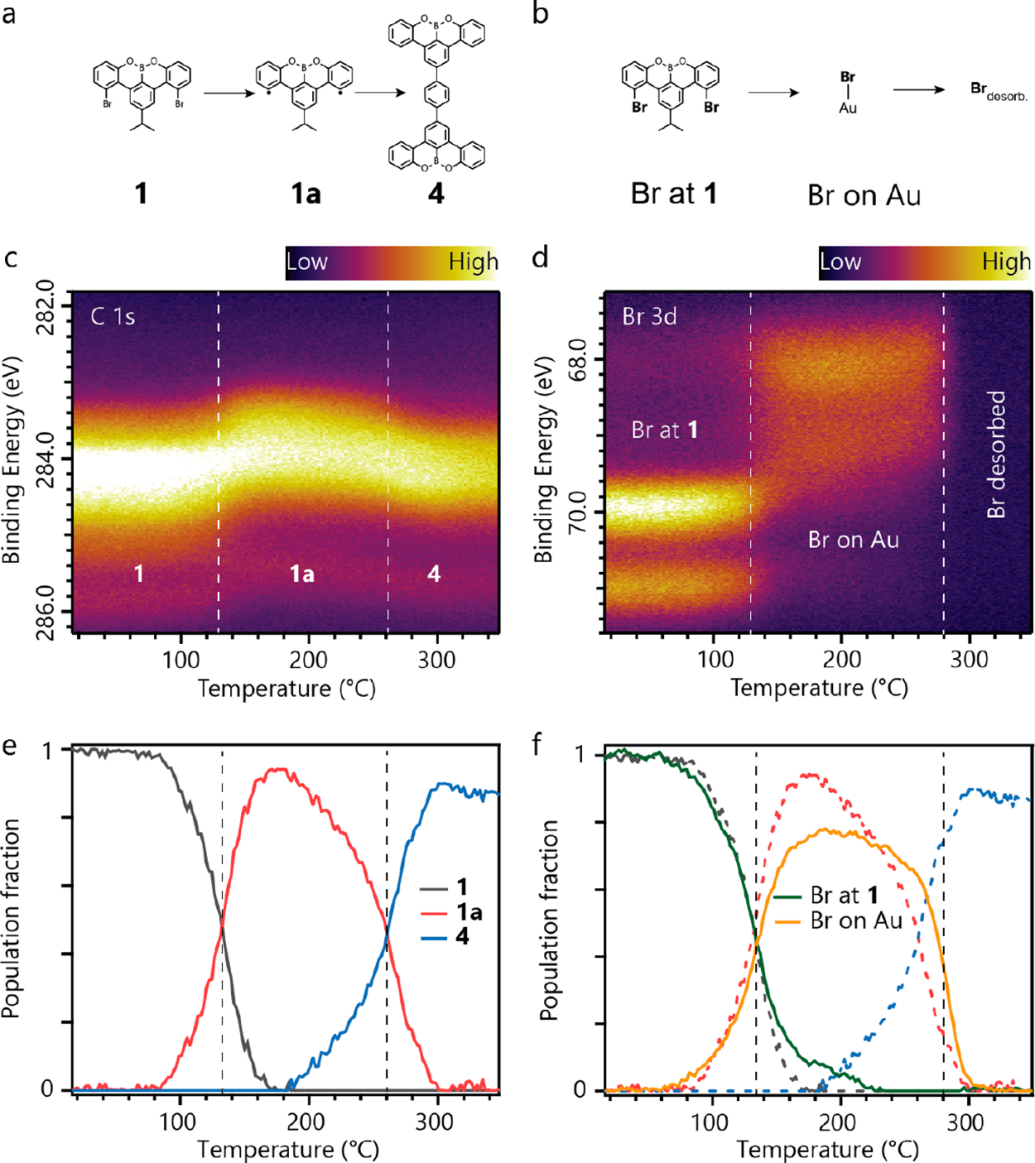
Kinetics of the reaction via TP-XPS: C 1s and Br 3d core level photoelectron spectra as a function of temperature track the progress of the reaction through the distinct stages indicated in (a) and (b) . (c) C 1s map, with the three distinct regions indicated by dashed white lines. (d) Br 3d map with the three regions indicated by dashed white lines. Desorption of bromine beyond 280 °C leads to the disappearance of the Br 3d doublet. (e,f) Population fraction curves of C 1s (e) and Br 3d (f) reveal how the population of the identified species evolves with temperature. C 1s population fraction curves are underlaid as dashed lines in (f). Vertical dashed lines mark the transition temperatures.

The frequent use of debrominative homocoupling in GNR synthesis^18^ makes the coexistence of Br and isopropyl substituents an opportunity to examine their interaction as a function of temperature. XPS spectra over the C 1s and Br 3d core levels measured at various temperatures provide this characterization (Figure 4). Chemical shifts in these core levels during heating with a constant rate of 0.1 °C/s give direct insights into the population fraction of different species in such thermally triggered reactions. We identify three regions with a dominant species in C 1s (corresponding to **1**, **1a** and **4**, Figure 4a) and Br 3d maps (corresponding to Br on **1**, Br chemisorbed on Au and desorbed into a vacuum, Figure 4b). Temperatures at which only a single surface species exists are identified, and XPS spectra at these temperatures are used to fit intermediate temperature spectra and obtain population fractions as a function of temperature^19,20^ (See Methods for fitting procedure).

From the population fraction curves in Figure 4e, we determine that the debromination of **1** to **1a** happens between 90–160 °C, and the formation of **4** between 180–300 °C, with half conversion at 130 and 260 °C for the two steps, respectively. A reduction in the total intensity of the C 1s signal is caused by desorption of some precursor molecules of **1** from the Au(111) surface (Figure 4c,e) as the starting coverage was slightly larger than that of a full monolayer of precursor molecules. As the sublimation temperature of the precursor is relatively low at 95 °C, we expect the precursors from the second layer to desorb, thus leaving a monolayer of molecules that are more strongly interacting with the Au surface. The TP-XPS measurements indicate that the formation of **4** starts at 180 °C, hence maintaining the sample at 200 °C for extended period of time would allow the reaction to complete, as seen in Figure 2f,g. Additionally, comparing Figure 4e and Figure 4f it is seen that the formation of **4** occurs in the presence of chemisorbed Br atoms. This suggests the Br presence^21^ does not significantly impact the fate of the isopropyl substituents toward [3 + 3] cycloaromatization. Additionally, these results provide useful insights into the design of molecules incorporating Br and isopropyl substitutions for future on-surface synthetic attempts.

## Conclusions

The [3 + 3] cycloaromatization of isopropyl substituents on arenes provides an efficient strategy for the synthesis of arylene-phenylene copolymers on Au surfaces. Such arylene-based copolymers, in general, find use in organic field-effect transistors, where a major limitation is the low charge-carrier mobility caused by short oligomers. Our results show that installing the isopropyl substituents on wide arene cores sterically prevents undesirable cross-linking reactions. Therefore, this strategy can be used to obtain long copolymer chains free of branching, based on the [3 + 3] cycloaromatization reaction. More generally, our demonstrated steric protection is widely applicable and can be used in on-surface synthesis to enhance the selectivity of other surface-catalyzed intermolecular reactions.

## Experimental Section

### Synthesis of **1**

See Supporting Information, section 1.

### STM and nc-AFM Experiments

The experiments were performed in ultrahigh vacuum (UHV) systems with a base pressure better than 5 × 10^–10^ mbar. The Au(111) single crystals (MaTeck GmbH) were cleaned by repeated cycles of Ar^+^ ion sputtering (1 keV) and subsequent annealing (400 °C < *T* < 450 °C). **1** was sublimated from a quartz crucible at ∼95 °C and deposited onto the cleaned surfaces (held at room temperature) *in situ* using a custom-made evaporator to achieve submonolayer coverages. The sample was subsequently annealed to the desired temperatures and kept at those temperatures for ∼30 min.

The sample was then cooled down and inserted into the liquid helium cooled STM (Scienta Omicron) operated at 4.7 K. The STM/nc-AFM measurements were performed with a tungsten tip placed on a qPlus tuning fork sensor.^22^ For nc-AFM measurements^23−25^ the qPlus sensor was excited at its resonance frequency of 26589 Hz (Q-factor 53000) and the shift in the frequency was tracked in the constant-height mode using Omicron Matrix electronics and HF2Li PLL by Zurich Instruments. The tip was functionalized with a single CO molecule at the tip apex picked up from the previously CO-dosed surface.^26^ Δ*z* is set to zero when the feedback loop is switched off. It is positive when the tip–surface distance is increased and negative when it is decreased.

### Computational Details

All DFT calculations were performed with the AiiDAlab platform^27^ based on AiiDA^28^ and the CP2K code.^29^ The surface–adsorbate systems were modeled within the repeated slab scheme, with the simulation cell consisting of 4 atomic layers of Au along the [111] direction. To suppress the Au(111) surface state, the bottom side of the slab was passivated by a layer of hydrogen atoms. 40 Å of vacuum was included in the simulation cell to decouple the system from its periodic replicas in the direction perpendicular to the surface. The electronic states were expanded with a TZV2P Gaussian basis set^30^ for C and H species and a DZVP basis set for Au species. A cutoff of 600 Ry was used for the plane-wave basis set. The frozen core electrons of the atoms were represented by norm-conserving Goedecker-Teter-Hutter pseudopotentials.^31^ We used the PBE parametrization for the generalized gradient approximation of the exchange-correlation functional^32^ and the D3 scheme proposed by Grimme^33^ to account for van der Waals interactions. The gold surface was modeled using a supercell, with its size ranging from 39.8 × 40.0 Å^2^ (corresponding to 896 Au atoms) to 35.38 × 35.74 Å^2^ (841 Au atoms) depending on the dimensions of the adsorbate. To obtain the equilibrium geometries, we kept the atomic positions of the bottom two layers of the slab fixed to the ideal bulk positions, and all other atoms were relaxed until the forces were lower than 0.005 eV/Å. For nc-AFM simulations with AiiDAlab, the equilibrium geometries and the electrostatic potential obtained with CP2K are used in combination with the probe particle code developed by Hapala.^34^

### Temperature-Programmed XPS Measurements

X-ray photoelectron spectroscopy (XPS) measurements were performed on the PEARL beamline^35^ of the SLS synchrotron radiation facility (Villigen, Switzerland), using linearly polarized radiation with photon energy of 425 eV. After molecule deposition at room temperature, the sample was transferred into the analysis chamber where the temperature-resolved XPS map was recorded during the in situ annealing of the sample at a rate of 0.1 °C/s. The XPS spectra were obtained in normal emission geometry, using a hemispherical electron analyzer equipped with a multichannel plate (MCP) detector. The XPS measurement was performed using the “fix” mode (snapshots of the C 1s level and Br 3d level), acquiring one spectrum each every 5 s with 50 eV pass energy. These spectra were plotted as a function of the temperature into TP-XPS maps. To obtain the population fraction curves, we averaged XPS spectra in a 10 °C window centered at the following temperatures: T1:20 °C (for both C 1s and Br 3d maps), T2:180 °C (for the C 1s map), 233 °C (for the Br 3d map), and T3:343 °C (for both C 1s and Br 3d maps). These six XPS spectra,shown in Supporting Information S5, are chosen to represent the six pure states shown in Figure 4a,b. We obtain the population fraction at intermediate temperatures by expressing the XPS spectra obtained at that temperature [XPS(T)] as a linear sum of the corresponding pure state XPS spectra. The obtained coefficients are thus equivalent to the population fractions. For example: for the C 1s map, consider T such that T1 < *T* < T2 where T1 = 20 °C and T2 = 180 °C. We calculate the following: XPS(T)= *n*(T) × XPS_Pure_(T1) + *m*(T) × XPS_Pure_(T2), where *n*(T) and *m*(T) are optimized with a linear fit. Thus, *n*(T) and *m*(T) are population fractions corresponding to **1** and **1a**. The population fraction of state at T1 is taken to be unity. The code for reproducing the analysis is shared alongside the raw data as a Jupyter notebook. Additionally, high-resolution XPS spectra obtained before and after the TP-XPS ramp are shown in Supporting Information S6.

## Data Availability

CCDC 2213680 (1) contains the supplementary crystallographic data for this paper. These data can be obtained free of charge via www.ccdc.cam.ac.uk/data_request/cif, or by emailing data_request@ccdc.cam.ac.uk, or by contacting The Cambridge Crystallographic Data Centre, 12 Union Road, Cambridge CB2 1EZ, UK; fax: + 44 1223 336033. The data that support the findings of this study are available at the Materials Cloud Platform (DOI: 10.24435/materialscloud:21-aj).

## Supporting Information Available

Synthesis details, experimental details, single-crystal structure, ^1^H and ^13^C NMR spectra (PDF)

## References

[ref1] Clair S.; de Oteyza D. G. Controlling a Chemical Coupling Reaction on a Surface: Tools and Strategies for On-Surface Synthesis. Chem. Rev. 2019, 119 (7), 4717–4776. 10.1021/acs.chemrev.8b00601. 30875199 PMC6477809

[ref2] Grill L.; Hecht S. Covalent On-Surface Polymerization. Nat. Chem. 2020, 12 (2), 115–130. 10.1038/s41557-019-0392-9. 31996811

[ref3] Wang T.; Fan Q.; Zhu J. Steering On-Surface Reactions by Kinetic and Thermodynamic Strategies. J. Phys. Chem. Lett. 2023, 14 (9), 2251–2262. 10.1021/acs.jpclett.3c00001. 36821589

[ref4] Kinikar A.; Di Giovannantonio M.; Urgel J. I.; Eimre K.; Qiu Z.; Gu Y.; Jin E.; Narita A.; Wang X.-Y.; Müllen K.; Ruffieux P.; Pignedoli C. A.; Fasel R. On-Surface Polyarylene Synthesis by Cycloaromatization of Isopropyl Substituents. Nat. Synth 2022, 1 (4), 289–296. 10.1038/s44160-022-00032-5.

[ref5] Fan Q.; Wang T.; Dai J.; Kuttner J.; Hilt G.; Gottfried J. M.; Zhu J. On-Surface Pseudo-High-Dilution Synthesis of Macrocycles: Principle and Mechanism. ACS Nano 2017, 11 (5), 5070–5079. 10.1021/acsnano.7b01870. 28419801

[ref6] Jacobse P. H.; van den Hoogenband A.; Moret M.-E.; Klein Gebbink R. J. M.; Swart I. Aryl Radical Geometry Determines Nanographene Formation on Au(111). Angew. Chem., Int. Ed. 2016, 55, 13052 10.1002/anie.201606440. 27632976

[ref7] Xu X.; Kinikar A.; Di Giovannantonio M.; Ruffieux P.; Müllen K.; Fasel R.; Narita A. On-Surface Synthesis of Dibenzohexacenohexacene and Dibenzopentaphenoheptaphene. BCSJ. 2021, 94 (3), 997–999. 10.1246/bcsj.20200382.

[ref8] Stolz S.; Di Giovannantonio M.; Urgel J. I.; Sun Q.; Kinikar A.; Borin Barin G.; Bommert M.; Fasel R.; Widmer R. Reversible Dehalogenation in On-Surface Aryl–Aryl Coupling. Angew. Chem., Int. Ed. 2020, 59 (33), 14106–14110. 10.1002/anie.202005443. 32338418

[ref9] DeJong M.; Price A. J. A.; Mårsell E.; Tom G.; Nguyen G. D.; Johnson E. R.; Burke S. A. Small Molecule Binding to Surface-Supported Single-Site Transition-Metal Reaction Centres. Nat. Commun. 2022, 13 (1), 7407 10.1038/s41467-022-35193-6. 36456555 PMC9715722

[ref10] Krull C.; Castelli M.; Hapala P.; Kumar D.; Tadich A.; Capsoni M.; Edmonds M. T.; Hellerstedt J.; Burke S. A.; Jelinek P.; Schiffrin A. Iron-Based Trinuclear Metal-Organic Nanostructures on a Surface with Local Charge Accumulation. Nat. Commun. 2018, 9 (1), 3211 10.1038/s41467-018-05543-4. 30097562 PMC6086834

[ref11] Pawlak R.; Meier T.; Renaud N.; Kisiel M.; Hinaut A.; Glatzel T.; Sordes D.; Durand C.; Soe W.-H.; Baratoff A.; Joachim C.; Housecroft C. E.; Constable E. C.; Meyer E. Design and Characterization of an Electrically Powered Single Molecule on Gold. ACS Nano 2017, 11 (10), 9930–9940. 10.1021/acsnano.7b03955. 28756663

[ref12] Boneschanscher M. P.; Hämäläinen S. K.; Liljeroth P.; Swart I. Sample Corrugation Affects the Apparent Bond Lengths in Atomic Force Microscopy. ACS Nano 2014, 8 (3), 3006–3014. 10.1021/nn500317r. 24559211

[ref13] Hapala P.; Kichin G.; Wagner C.; Tautz F. S.; Temirov R.; Jelínek P. Mechanism of High-Resolution STM/AFM Imaging with Functionalized Tips. Phys. Rev. B 2014, 90 (8), 085421 10.1103/PhysRevB.90.085421. 25494078

[ref14] Hapala P.; Yakutovich A. ProbeParticleModel, 2016. https://github.com/ProkopHapala/ProbeParticleModel. (accessed 2021-10-11).

[ref15] Schuler B.; Liu W.; Tkatchenko A.; Moll N.; Meyer G.; Mistry A.; Fox D.; Gross L. Adsorption Geometry Determination of Single Molecules by Atomic Force Microscopy. Phys. Rev. Lett. 2013, 111 (10), 106103 10.1103/PhysRevLett.111.106103. 25166684

[ref16] Biswas K.; Urbani M.; Sánchez-Grande A.; Soler-Polo D.; Lauwaet K.; Matěj A.; Mutombo P.; Veis L.; Brabec J.; Pernal K.; Gallego J. M.; Miranda R.; Écija D.; Jelínek P.; Torres T.; Urgel J. I. Interplay between π-Conjugation and Exchange Magnetism in One-Dimensional Porphyrinoid Polymers. J. Am. Chem. Soc. 2022, 144 (28), 12725–12731. 10.1021/jacs.2c02700. 35817408 PMC9305978

[ref17] Gao H.-Y.; Wagner H.; Zhong D.; Franke J.-H.; Studer A.; Fuchs H. Glaser Coupling at Metal Surfaces. Angew. Chem., Int. Ed. 2013, 52 (14), 4024–4028. 10.1002/anie.201208597. 23424176

[ref18] Houtsma R. S. K.; de la Rie J.; Stohr M. Atomically Precise Graphene Nanoribbons: Interplay of Structural and Electronic Properties. Chem. Soc. Rev. 2021, 50 (11), 6541–6568. 10.1039/D0CS01541E. 34100034 PMC8185524

[ref19] Di Giovannantonio M.; El Garah M.; Lipton-Duffin J.; Meunier V.; Cardenas L.; Fagot Revurat Y.; Cossaro A.; Verdini A.; Perepichka D. F.; Rosei F.; Contini G. Insight into Organometallic Intermediate and Its Evolution to Covalent Bonding in Surface-Confined Ullmann Polymerization. ACS Nano 2013, 7 (9), 8190–8198. 10.1021/nn4035684. 23987501

[ref20] Di Giovannantonio M.; Tomellini M.; Lipton-Duffin J.; Galeotti G.; Ebrahimi M.; Cossaro A.; Verdini A.; Kharche N.; Meunier V.; Vasseur G.; Fagot-Revurat Y.; Perepichka D. F.; Rosei F.; Contini G. Mechanistic Picture and Kinetic Analysis of Surface-Confined Ullmann Polymerization. J. Am. Chem. Soc. 2016, 138 (51), 16696–16702. 10.1021/jacs.6b09728. 27958750

[ref21] Pham T. A.; Song F.; Nguyen M.-T.; Li Z.; Studener F.; Stöhr M. Comparing Ullmann Coupling on Noble Metal Surfaces: On-Surface Polymerization of 1,3,6,8-Tetrabromopyrene on Cu(111) and Au(111). Chemistry – A European Journal 2016, 22 (17), 5937–5944. 10.1002/chem.201504946. 26879625

[ref22] Giessibl F. J. Atomic Resolution on Si(111)-(7 × 7) by Noncontact Atomic Force Microscopy with a Force Sensor Based on a Quartz Tuning Fork. Appl. Phys. Lett. 2000, 76 (11), 1470–1472. 10.1063/1.126067.

[ref23] Gross L.; Mohn F.; Moll N.; Liljeroth P.; Meyer G. The Chemical Structure of a Molecule Resolved by Atomic Force Microscopy. Science 2009, 325 (5944), 1110 10.1126/science.1176210. 19713523

[ref24] Gross L. Recent Advances in Submolecular Resolution with Scanning Probe Microscopy. Nat. Chem. 2011, 3 (4), 273–278. 10.1038/nchem.1008. 21430684

[ref25] Gross L.; Schuler B.; Pavliček N.; Fatayer S.; Majzik Z.; Moll N.; Peña D.; Meyer G. Atomic Force Microscopy for Molecular Structure Elucidation. Angew. Chem., Int. Ed. 2018, 57 (15), 3888–3908. 10.1002/anie.201703509. 29485190

[ref26] Bartels L.; Meyer G.; Rieder K.-H.; Velic D.; Knoesel E.; Hotzel A.; Wolf M.; Ertl G. Dynamics of Electron-Induced Manipulation of Individual CO Molecules on Cu(111). Phys. Rev. Lett. 1998, 80 (9), 2004–2007. 10.1103/PhysRevLett.80.2004.

[ref27] Yakutovich A. V.; Eimre K.; Schütt O.; Talirz L.; Adorf C. S.; Andersen C. W.; Ditler E.; Du D.; Passerone D.; Smit B.; Marzari N.; Pizzi G.; Pignedoli C. A. AiiDAlab – an Ecosystem for Developing, Executing, and Sharing Scientific Workflows. Comput. Mater. Sci. 2021, 188, 110165 10.1016/j.commatsci.2020.110165.

[ref28] Pizzi G.; Cepellotti A.; Sabatini R.; Marzari N.; Kozinsky B. AiiDA: Automated Interactive Infrastructure and Database for Computational Science. Comput. Mater. Sci. 2016, 111, 218–230. 10.1016/j.commatsci.2015.09.013.

[ref29] Hutter J.; Iannuzzi M.; Schiffmann F.; VandeVondele J. Cp2k: Atomistic Simulations of Condensed Matter Systems. Wiley Interdisciplinary Reviews: Computational Molecular Science 2014, 4 (1), 15–25. 10.1002/wcms.1159.

[ref30] VandeVondele J.; Hutter J. Gaussian Basis Sets for Accurate Calculations on Molecular Systems in Gas and Condensed Phases. J. Chem. Phys. 2007, 127 (11), 114105 10.1063/1.2770708. 17887826

[ref31] Goedecker S.; Teter M.; Hutter J. Separable Dual-Space Gaussian Pseudopotentials. Phys. Rev. B 1996, 54 (3), 1703–1710. 10.1103/PhysRevB.54.1703. 9986014

[ref32] Perdew J. P.; Burke K.; Ernzerhof M. Generalized Gradient Approximation Made Simple. Phys. Rev. Lett. 1996, 77 (18), 3865–3868. 10.1103/PhysRevLett.77.3865. 10062328

[ref33] Grimme S.; Antony J.; Ehrlich S.; Krieg H. A Consistent and Accurate Ab Initio Parametrization of Density Functional Dispersion Correction (DFT-D) for the 94 Elements H-Pu. J. Chem. Phys. 2010, 132 (15), 154104 10.1063/1.3382344. 20423165

[ref34] Hapala P.; Kichin G.; Wagner C.; Tautz F. S.; Temirov R.; Jelínek P. Mechanism of High-Resolution STM/AFM Imaging with Functionalized Tips. Phys. Rev. B 2014, 90 (8), 085421 10.1103/PhysRevB.90.085421. 25494078

[ref35] Muntwiler M.; Zhang J.; Stania R.; Matsui F.; Oberta P.; Flechsig U.; Patthey L.; Quitmann C.; Glatzel T.; Widmer R.; Meyer E.; Jung T. A.; Aebi P.; Fasel R.; Greber T. Surface Science at the PEARL Beamline of the Swiss Light Source. J. Synchrotron Rad, J. Synchrotron Radiat 2017, 24 (1), 354–366. 10.1107/S1600577516018646. PMC5182030 28009578

