# Supplementary material for: Sterically Selective [3 + 3] Cycloaromatization in the On-Surface Synthesis of Nanographenes
Source: ACS Nanosci Au. 2023 Dec 22;4(2):128–35. doi: 10.1021/acsnanoscienceau.3c00062 (PMC11027121; doi:10.1021/acsnanoscienceau.3c00062)
Supplement: Supplementary file 1 — ng3c00062_si_001.pdf [file ng3c00062_si_001.pdf]

Supporting Information

Sterically selective [3+3] cycloaromatization in the on-surface synthesis of nanographenes

Amogh Kinikar°[1], Xiao-Ye Wang°[2,3], Marco Di Giovannantonio°[1,7], José I. Urgel°[1,8], Pengcai Liu[3], Kristjan Eimre[1], Carlo A. Pignedoli*[1], Samuel Stolz[1,6,8], Max Bommert[1], Shantanu Mishra[1,10], Qiang Sun[1,11], Roland Widmer[1], Zijie Qiu[2,12], Akimitsu Narita[2], Klaus Müllen*[2, 4], Pascal Ruffieux[1], and Roman Fasel*[1, 5]

[1]Empa, Swiss Federal Laboratories for Materials Science and Technology, 8600 Dübendorf (Switzerland)
[2]Max Planck Institute for Polymer Research, 55128 Mainz (Germany)

[3]State Key Laboratory of Elemento-Organic Chemistry, College of Chemistry, Nankai University, Tianjin 300071 (China)

[4]Department of Chemistry, Johannes Gutenberg-Universität Mainz, 55128 Mainz (Germany)
[5]Department of Chemistry, Biochemistry and Pharmaceutical Sciences, University of Bern, 3012 Bern (Switzerland)
[6]Institute of Condensed Matter Physics, Station 3, EPFL, 1015 Lausanne (Switzerland)
Present address:

[7]Istituto di Struttura della Materia-CNR (ISM-CNR), via Fosso del Cavaliere 100, Roma 00133 (Italy)
[8]IMDEA Nanoscience, C/Faraday 9, Campus de Cantoblanco, Madrid 28049 (Spain)

[9] Department of Physics, University of California at Berkeley, Berkeley, California 94720 (USA)

[10]IBM Research — Zurich, Rüschlikon, 8803 (Switzerland)

[11]Materials Genome Institute, Shanghai University, 200444 Shanghai (China)

[12]School of Science and Engineering, Shenzhen Institute of Aggregate Science and Technology, The Chinese University of Hong Kong, Shenzhen, Guangdong 518172 (China)

° These authors contributed equally
* Address correspondence to these authors

CONTENTS

1. Synthesis Details
2. Experimental Details
3. Single-Crystal Structure
4. ^{1}H and ^{13}C NMR Spectra
5. References

1. SYNTHESIS DETAILS

General Methods. All commercial chemicals were used as received without further purification unless otherwise noted. All yields given refer to isolated yields. Nuclear magnetic resonance (NMR) spectra were recorded on an AVANCE 300 MHz or 400 MHz Bruker spectrometer. ^{1}H NMR chemical shifts were referenced to CHD_2COCD_3 (2.050 ppm) or $CDHCl_2$ (5.320 ppm). ^{13}C NMR chemical shifts were referenced to CD_3COCD_3 (29.80 ppm) or CD_2Cl_2 (54.00 ppm). The following abbreviations were used for multiplicities: s = singlet, d = doublet, t = triplet, sept = septet, m = multiplet. High-resolution mass spectrometry (HRMS) was performed on a Varian 7.0T FTMS by matrix-assisted laser desorption/ionization (MALDI) with 2,5-dihydroxybenzoic acid or tetracyanoquinodimethane (TCNQ) as the matrix.

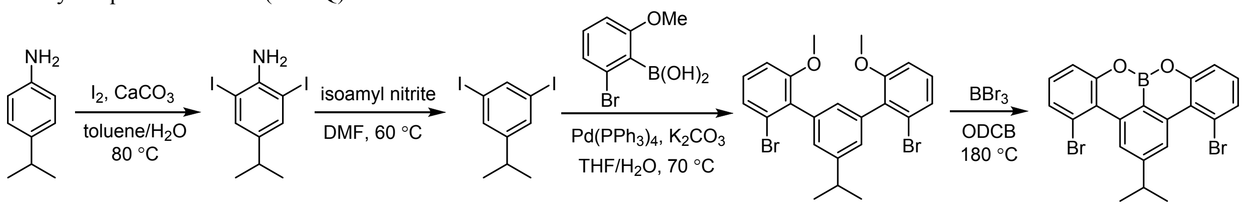

Scheme S1. Synthetic route to precursor 1.

Synthetic Procedures and Data

3,5-Diiodo-1-isopropylbenzene (**S3**) was synthesized according to the reported procedure.[1]

2,2''-Dibromo-5'-isopropyl-6,6''-dimethoxy-1,1':3',1''-terphenyl (S4). To a Schlenk tube under argon was added compound **S3** (186 mg, 0.500 mmol), 2-bromo-6-methoxyphenyl boronic acid (242 mg, 1.05 mmol), $Pd(PPh_3)_4$ (35 mg, 0.030 mmol), and K_2CO_3 (276 mg, 2.00 mmol). THF (20 mL) and H_2O (1 mL) were then added to the mixture, which was stirred at 70 °C for 48 h. After cooling down to room temperature, the mixture was extracted with EtOAc for three times. The combined organic layers were washed with brine and dried over $MgSO_4$. After removal of the solvent under reduced pressure, the residue was purified by column chromatography over silica gel (eluent: hexane/DCM = 2 : 1) to afford 92 mg (yield: 38%) of compound **S4** as a light yellow solid. ^{1}H NMR (400 MHz, $(CD_3)_2CO$, 297 K, ppm) δ 7.33 – 7.21 (m, 4H), 7.15 – 7.07 (m, 4H), 6.93 (d, J = 12.4 Hz, 1H), 3.74 (s, 6H), 2.99 (sept, J = 6.9 Hz, 1H), 1.30 (d, J = 7.0 Hz, 6H). ^{13}C NMR (101 MHz, $(CD_3)_2CO$, 297 K, ppm) δ 159.13, 148.11, 137.67, 137.32, 132.80, 130.69, 130.38, 128.19, 125.62, 111.45, 56.41, 34.64, 24.28. HRMS (MALDI) *m/z*: Calcd. for $C_{23}H_{22}Br_2NaO_2$: 510.9884; Found: 510.9882 $[M + Na]^+$.

4,13-Dibromo-2-isopropyl-8,9-dioxa-8a-borabenzo[*fg*]tetracene (1). To a solution of compound **S4** (76 mg, 0.16 mmol) in anhydrous 1,2-dichlorobenzene (10 mL) was added BBr_3 (1.0 M in heptane, 0.62 mL, 0.62 mmol) at room temperature under argon. The reaction mixture was stirred at 180 °C for 12 h. After quenching with methanol, the solvent was removed under reduced pressure. The residue was purified by column chromatography over silica gel (eluent: DCM/MeOH = 20 : 1) and further recrystallization from DCM/MeOH to afford 61 mg (81%) of compound **1** as a white solid. ^{1}H NMR (300 MHz, CD_2Cl_2, 297 K, ppm) δ 9.12 (s, 2H), 7.61 (dd, J = 7.9, 1.4 Hz, 2H), 7.40 (dd, J = 8.2, 1.4 Hz, 2H), 7.25 (t, J = 8.0 Hz, 2H), 3.22 (sept, J = 6.9 Hz, 1H), 1.43 (d, J = 6.9 Hz, 6H). ^{13}C NMR (75 MHz, CD_2Cl_2, 297 K, ppm) δ 153.82, 153.78, 139.19, 131.23, 129.64, 125.95, 124.05, 120.80, 120.40, 36.15, 24.29. HRMS (MALDI) *m/z*: Calcd. for $C_{21}H_{15}BBr_2O_2$: 467.9532; Found: 467.9530 M^+.

2. EXPERIMENTAL DETAILS

S1: Statistical analysis of the products on the surface

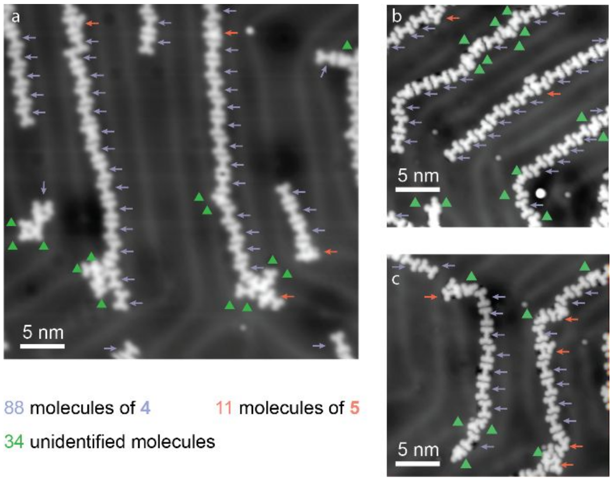

Figure S1: a-c STM image with the different products indicated. The molecules on the surface correspond to the reaction yield of 243 precursors. 176 of these have successfully undergone the reaction to yield 88 molecules of **4**. Additionally, each molecule of **5**, also contains two precursor units that have formed **4**, and a third molecule that has attacked the radical site generated by debromination. Together these correspond to a yield of 77 % ± 6%. 34 molecules could not be identified. There are no reaction products with three molecules reacting at the isopropyl substituent site. (Tunneling parameters: **a** : V = -0.1 V, I = 150 pA and **b**,**c** : V = -0.1 V, I = 100).

S2: Apparent distortion in the nc-AFM image as a function of tip height

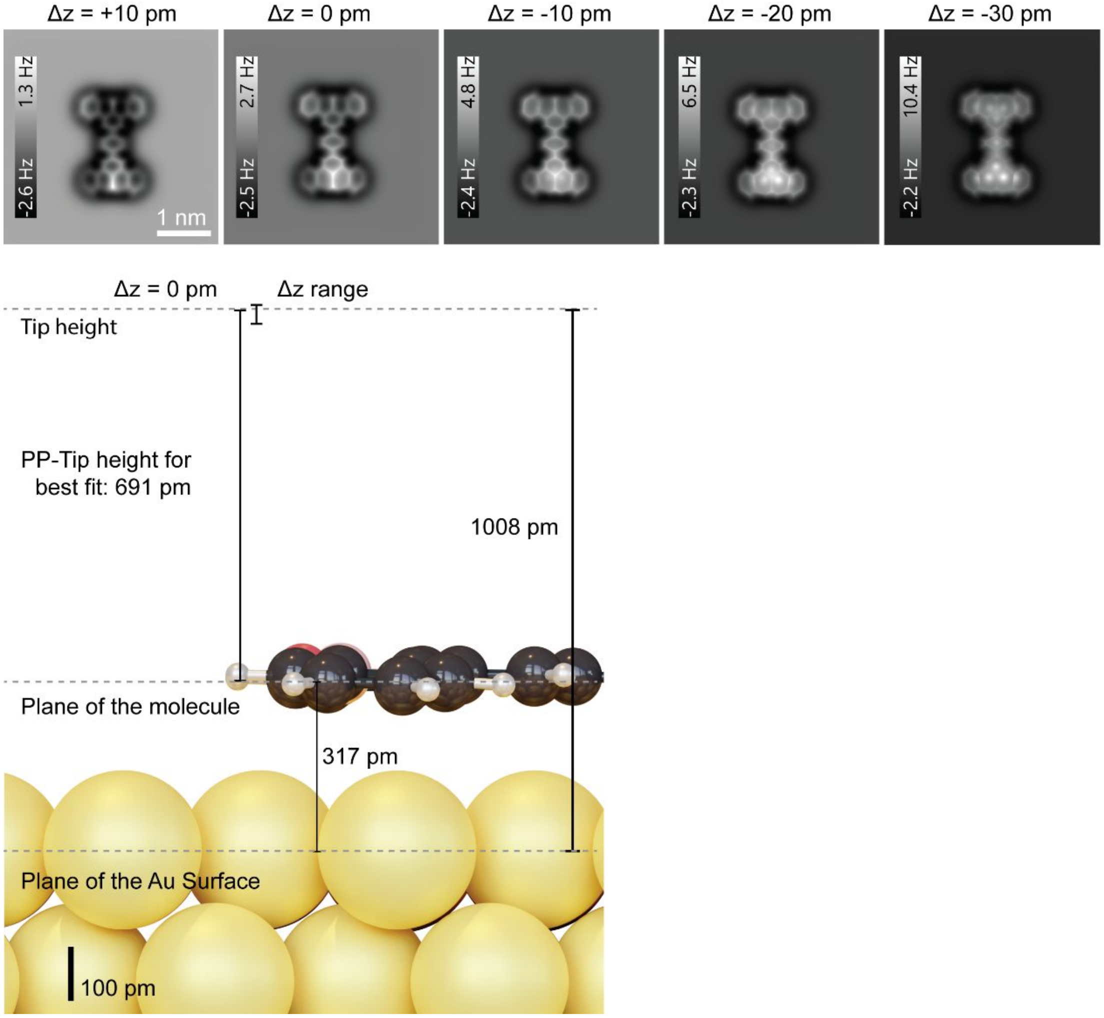

Figure S2: Simulated nc-AFM images reproduce the widening of the phenylene rings. The adsorbed CO molecule on the nc-AFM tip is flexible and can bend. When the nc-AFM tip is positioned over the edge of a molecule the resulting force on the CO molecule is asymmetric leading to the apparent stretching of the molecular edges, with this apparent stretching increasing with the flexibility at the adsorbed CO-tip junction[2]. A phenylene ring essentially consist only of edges and therefore it appears wider in the nc-AFM image. The edge related distortion is highly sensitive to the probing distance of the tip, and on the flexibility of the CO-tip junction, thus it depends on the unique atomic-level details of the adsorbed CO molecule and the apex of the metallic tip. However, it becomes more apparent (the phenylene rings appears wider) the closer the tip is to the adsorbed molecule. By performing the simulation at different heights above the surface, we can reproduce this distortion in good qualitative agreement.

S3: Structural characterization of 5

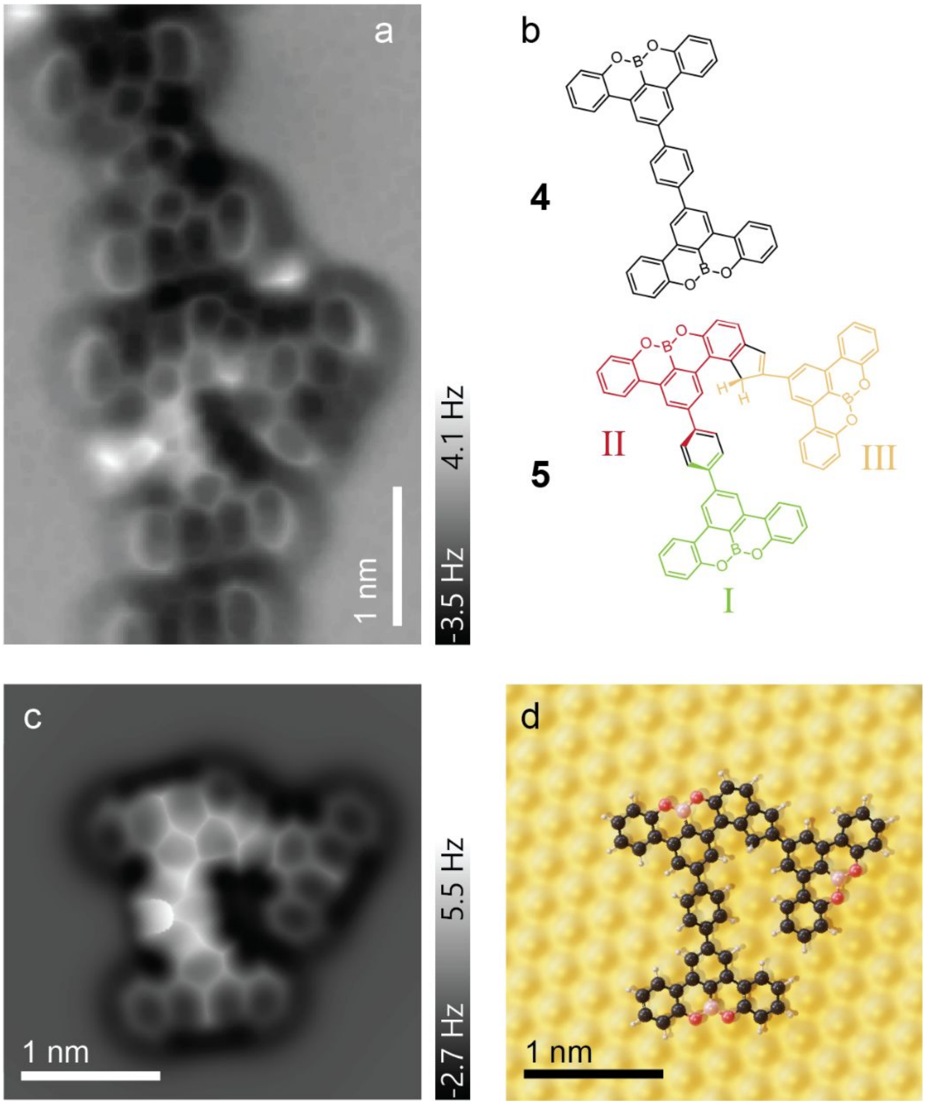

Figure S3: The reaction between a radical site and the isopropenyl group leads to the formation of 5. **a**, Bond resolved nc-AFM image acquired over molecule of **4** and **5** (Feedback switched off over the Au surface at -5 mV, 100 pA. The tip was lifted up by Δz = 252 pm and the frequency shift was measured in constant height mode). **b**, Chemical structure assigned based on **a**. Two molecules, labelled I and II, have successfully undergone cycloaromatization, to form a phenylene ring. This phenylene ring, when compared to the molecule of **4** above is twisted out of plane (indicated by wedge bonds) due to the presence of the third molecule (labelled III) which has attacked the radical site of II. **c**-**d**, Simulated nc-AFM image (**c**) using the PP model acquired on the optimized geometry of **5** on Au(111) (**d**) is in excellent agreement with the experiment.

S4: Reaction steps in the formation of 5

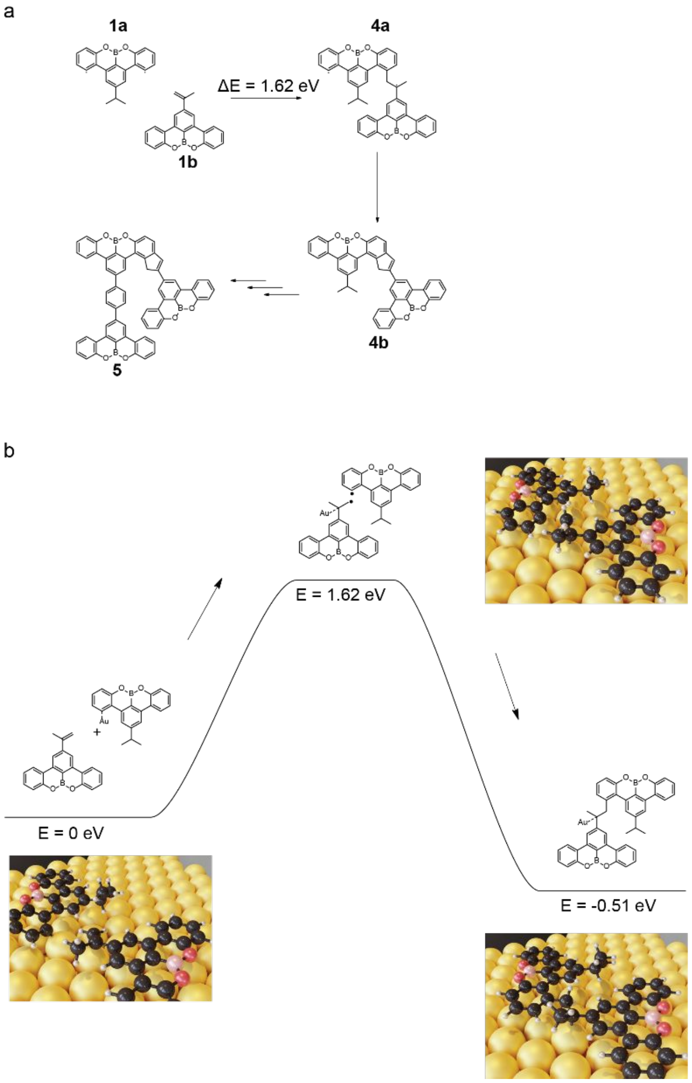

Figure S4: Mechanistic insights into the formation of the side product 5. **a**, Chemical schematic showing a plausible route towards the formation of **5**. **b**, The proposed route to **5** is intriguing because it involves an interaction between the σ-radical formed by debromination and a cycloaromatization intermediate such as the isopropenyl substituent in **1b**. DFT optimized constrained geometry calculations[3] were performed on this critical step to obtain the energy barrier for the coupling of the CH_2 site in **1b** with the radical site in **1a**. The resulting value of 1.65 eV is similar the DFT calculated barrier for the [3+3] cycloaromatization: 1.6 eV[3].

S5: Spectra from the TP-XPS map corresponding to the pure states

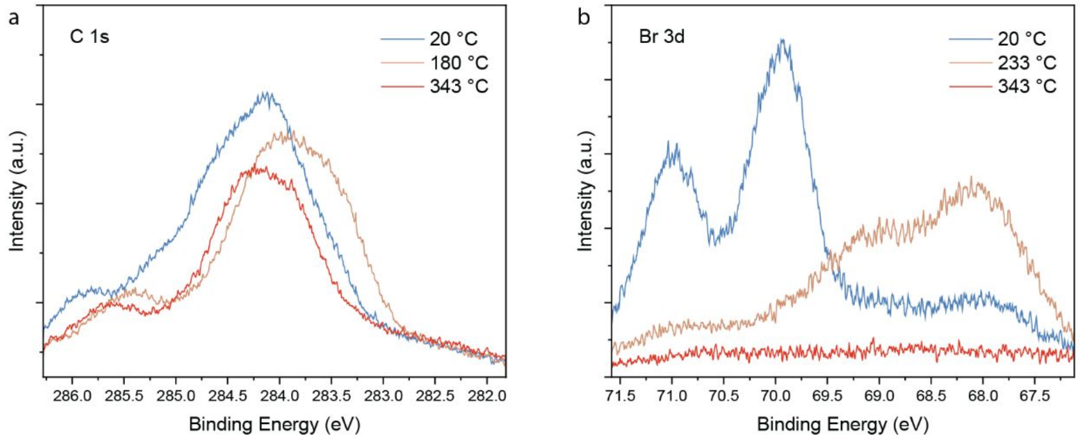

Figure S5: The XPS spectra over C 1s (**a**) and Br 3d (**b**) core levels corresponding to pure states. These spectra were obtained by averaging the TP-XPS line profiles over a 10 °C window centered at the indicated temperatures, to improve the signal-to-noise ratio.

S6: High-resolution XPS spectra

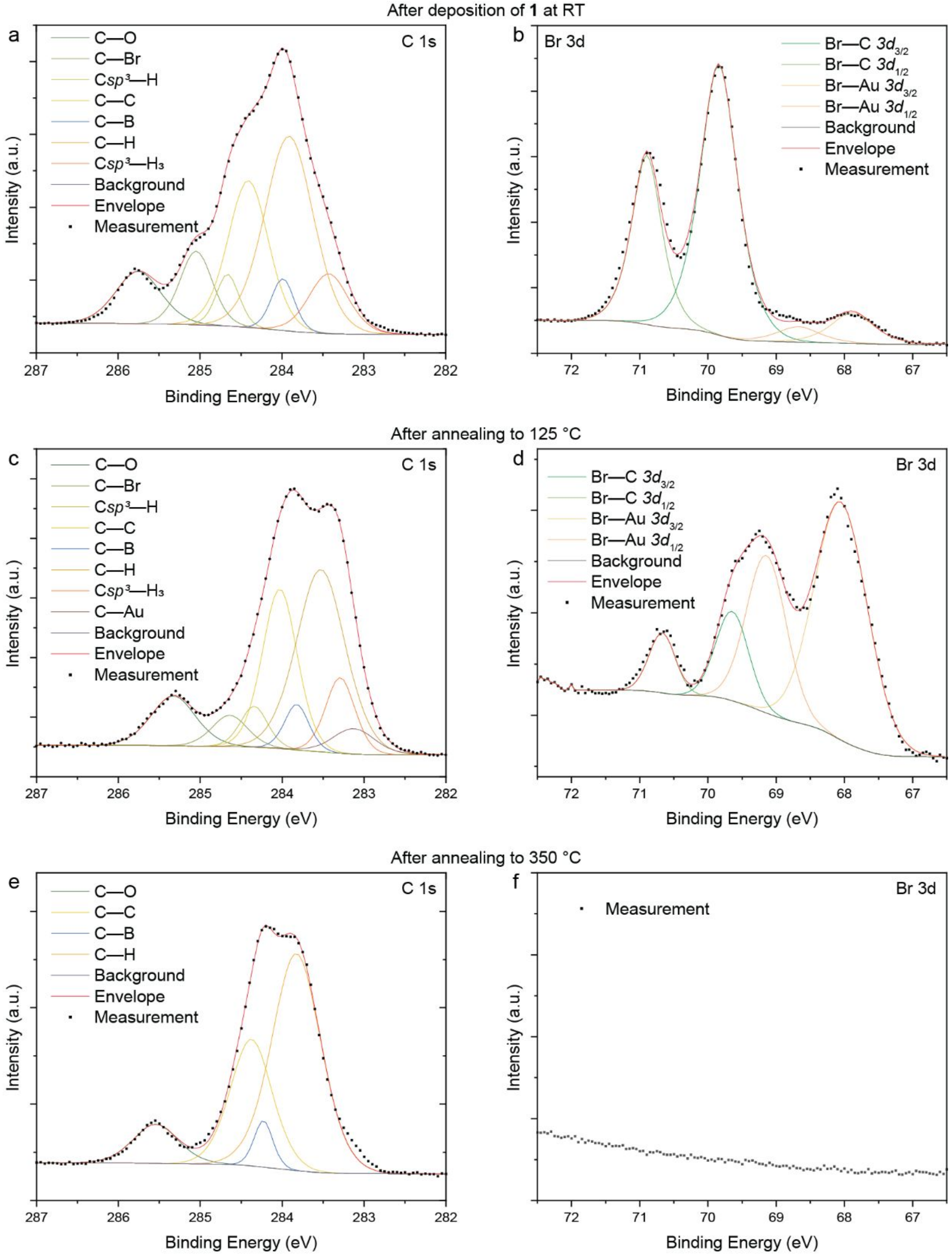

Figure S6: Component analysis of high-resolution XPS Spectra. The high-resolution XPS (HR-XPS) spectra in panels **a**, **b**, **e**, and **f** were obtained before and after the TP-XPS measurements reported in Fig. 3 in the main text. Another sample was prepared, and annealed to 125 °C (**c**, **d**). A component analysis of the HR-XPS spectra was performed using CasaXPS[4] A Shirley background was subtracted from each spectrum (a-e). A pseudo-Voigt function, i.e. a linear combination of a Lorentzian and a Gaussian function, was used to model the individual peaks. The area under the components was constrained to reflect the stoichiometry of the different carbon atoms in **1** (spectrum in **a**), organometallic dimers (spectrum in **c**), and in

4 (spectrum in **e**). With these constrains, the fitted curves are in reasonable agreement with the measured data. The component analysis of the Br 3d peaks is more straightforward as only two states are expected before the bromine atoms desorb, chemically bonded to a carbon atom (in **1**) or chemisorbed on the Au(111) surface. The peaks were similarly modeled using pseudo-Voigt functions. The area under a $3d_{1/2}$ component in a 3d doublet was constrained to be half of the area under the corresponding $3d_{3/2}$ component. We note the signatures of chemisorbed Br in **b**. This suggests that the substrate did not completely cool down to room temperature after cleaning, already triggering a partial debromination of the molecules being deposited. However, 90% of the molecules are still intact and therefore our analysis remains valid.

		C□—O	C□—Br	C*sp3*□—H	C□—C	C□—B	C□—H	C*sp3*□—H_3	C□—Au
RT	Position (eV)	285.76	285.05	284.19	284.52	283.96	283.91	283.43	□—
	FWHM (eV)	0.65	0.49	0.34	0.53	0.32	0.69	0.56	□—
125 ˚C	Position (eV)	285.33	284.64	284.34	284.03	283.82	283.53	283.29	283.13
	FWHM (eV)	0.64	0.51	0.39	0.5	0.35	0.7	0.42	0.6
350 ˚C	Position (eV)	285.54	□—	□—	284.38	284.23	283.83	□—	□—
	FWHM (eV)	0.65	□—	□—	0.6	0.27	0.7	□—	□—

Table S1: Fitting parameters for C 1s components.

		Br□—C *$3d_{1/2}$*	Br□—C *$3d_{3/2}$*	Br□—Au *$3d_{1/2}$*	Br□—Au *$3d_{3/2}$*
RT	Position (eV)	69.84	70.9	67.88	68.67
	FWHM (eV)	0.65	0.52	0.73	0.73
125 ˚C	Position (eV)	68.05	69.14	69.64	70.67
	FWHM (eV)	0.92	0.69	0.57	0.45

Table S2: Fitting parameters for Br 3d components.

3. SINGLE-CRYSTAL STRUCTURE

Single-crystal data of compound 1 **(CCDC 2213680)**

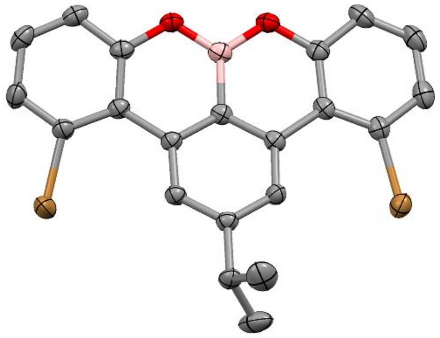

Figure S7. Single-crystal structure of compound 1. Thermal ellipsoids are shown at 50% probability. The hydrogen atoms are omitted for clarity.

Crystal data

formula	$C_{21}H_{15}BBr_2O_2$		
molecular weight	469.96 $gmol^{-1}$		
absorption	μ = 4.575 mm^{-1} corrected with 6 crystal faces		
transmission	T_{min} = 0.1889, T_{max} = 0.3629		
crystal size	0.27 x 0.31 x 0.50 mm^3 colorless block		
space group	P -1 (triclinic)		
lattice parameters	a = 7.9377(4)Å	α = 82.550(3)°	
(calculate from	b = 14.9931(6)Å	ß = 87.808(3)°	
17141 reflections with	c = 15.4854(6)Å	γ = 76.516(3)°	
2.0° < θ < 28.4°)	V = 1776.94(14)$Å^3$	z = 4	F(000) = 928
temperature	-80°C		
density	d_{xray} = 1.757 gcm^{-3}		

Data collection

diffractometer	STOE IPDS 2T
radiation	Mo-K_α Graphitmonochromator
Scan – type	ω scans
Scan – width	1°
Scan –range	2° ≤ θ < 28.3°
	-10 ≤ h ≤ 8 -19 ≤ k ≤ 19 -20 ≤ l ≤ 20
number of reflections:	
measured	15981
unique	8755 (R_{int} = 0.0315)
observed	6988 (|F|/σ(F) > 4.0)

Data correction, structure solution and refinement

corrections	Lorentz and polarization correction.
Structure solution	Program: SIR-2004 (Direct methods)
refinement	Program: SHELXL-2014 (full matrix). 474 refined parameters, weighting scheme: $w=1/[\sigma^2(F_o^2) + (0.0171*P)^2+6.34*P]$ with $(Max(F_o^2,0)+2*F_c^2)/3$. H-atoms at calculated positions and refined with isotropic displacement parameters, non H- atoms refined anisotropically.

R-values	wR2 = 0.1045 (R1 = 0.0438 for observed reflections, 0.0632 for all reflections)
goodness of fit	S = 1.120
maximum deviation of parameters	0.001 * e.s.d
maximum peak height in diff. Fourier synthesis	1.66, -1.46 eÅ^{-3}
remark	structure contains two independent molecules

4. ^{1}H AND ^{13}C NMR SPECTRA

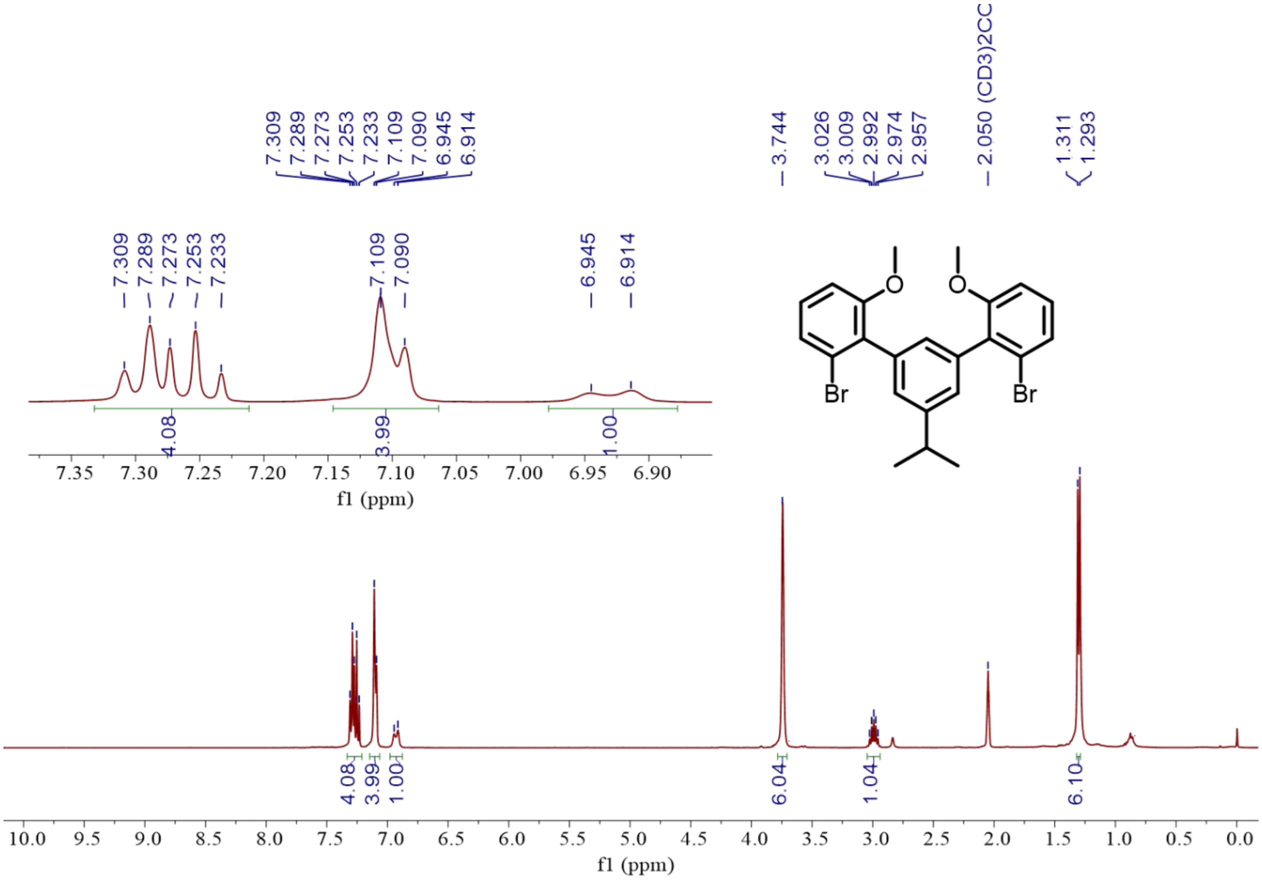

Figure S8. ^{1}H NMR spectrum of compound S4 (400 MHz, CD_3COCD_3, 297 K).

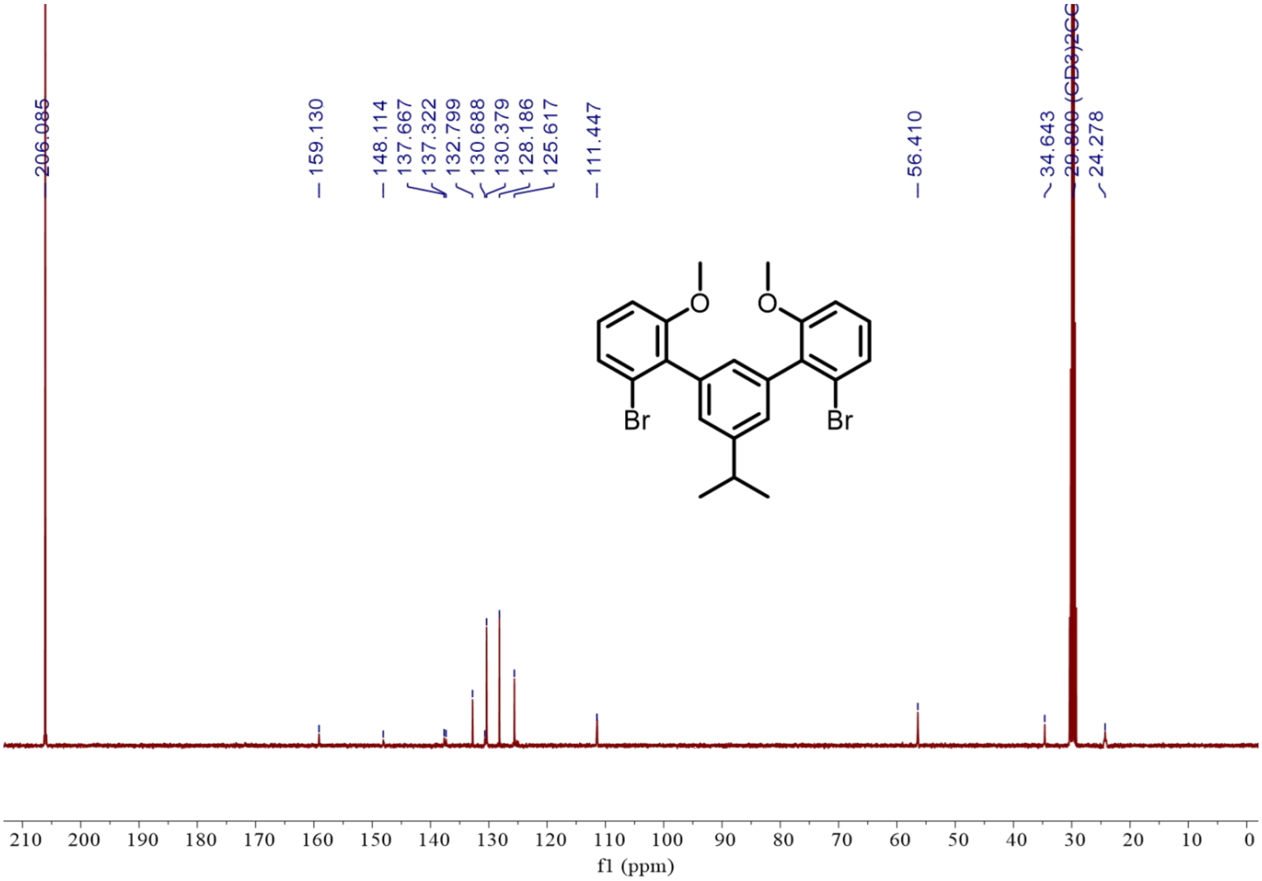

Figure S9. ^{13}C NMR spectrum of compound S4 (101 MHz, CD_3COCD_3, 297 K).

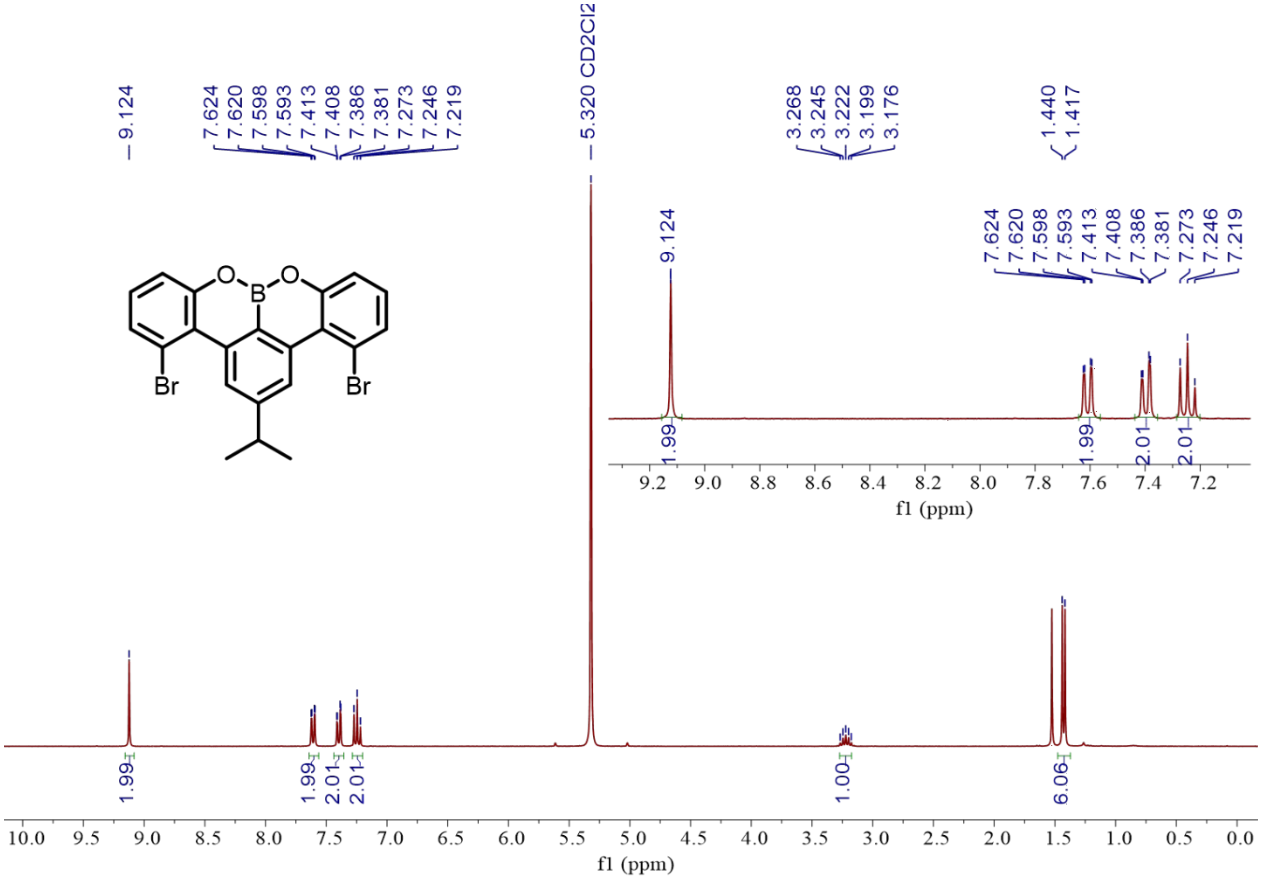

Figure S10. ^{1}H NMR spectrum of compound 1 (300 MHz, CD_2Cl_2, 297 K).

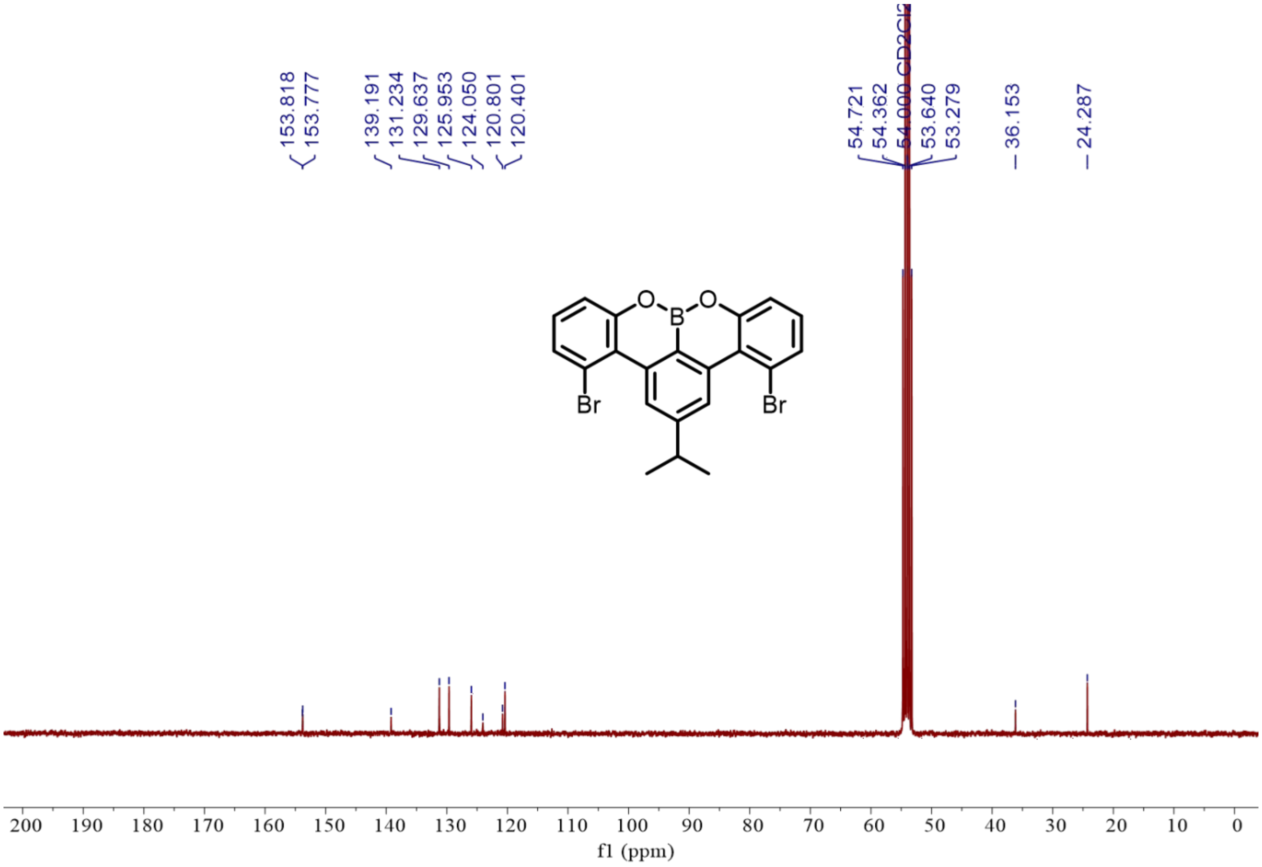

Figure S11. ^{13}C **NMR spectrum of compound 1 (75 MHz,** CD_2Cl_2**, 297 K).**

5. REFERENCES

1. Succaw, G. L., Weakley, T. J. R., Han, F. & Doxsee, K. M. Crystal Engineering with Bis(thiourea) Derivatives. *Cryst. Growth Des.* **5**, 2288–2298 (2005).

2. Boneschanscher, M. P., Hämäläinen, S. K., Liljeroth, P. & Swart, I. Sample Corrugation Affects the Apparent Bond Lengths in Atomic Force Microscopy. *ACS Nano* **8**, 3006–3014 (2014).

3. Kinikar, A. *et al.* On-surface polyarylene synthesis by cycloaromatization of isopropyl substituents. *Nat. Synth.* **1**, 289–296 (2022).

4. Fairley, N. *et al.* Systematic and collaborative approach to problem solving using X-ray photoelectron spectroscopy. *Appl. Surf. Sci. Adv.* **5**, 100112 (2021).
